# Aptamer-siRNA Chimeras: Discovery, Progress, and Future Prospects

**DOI:** 10.3390/biomedicines5030045

**Published:** 2017-08-09

**Authors:** Sven Kruspe, Paloma H. Giangrande

**Affiliations:** 1 Internal Medicine, University of Iowa, Iowa City, IA 52242, USA; sven-kruspe@uiowa.edu; 2 Molecular & Cellular Biology Program, Holden Comprehensive Cancer Center, Radiation Oncology, Abboud Cardiovascular Research Center, and Environmental Health Sciences Research Center (EHSRC), University of Iowa, Iowa City, IA 52242, USA

**Keywords:** aptamers, RNA interference, targeted therapy

## Abstract

Synthetic nucleic acid ligands (aptamers) have emerged as effective delivery tools for many therapeutic oligonucleotide-based drugs, including small interfering RNAs (siRNAs). In this review, we summarize recent progress in the aptamer selection technology that has made possible the identification of cell-specific, cell-internalizing aptamers for the cell-targeted delivery of therapeutic oligonucleotides. In addition, we review the original, proof-of-concept aptamer-siRNA delivery studies and discuss recent advances in aptamer-siRNA conjugate designs for applications ranging from cancer therapy to the development of targeted antivirals. Challenges and prospects of aptamer-targeted siRNA drugs for clinical development are further highlighted.

## 1. Introduction

More than 100 years ago, Paul Ehrlich (Nobel Prize Laureate in 1908) published the concept of a “magic bullet”, a tailored drug that, based on Ehrlich’s concept of cell-surface receptors, could home into the diseased cell without affecting the surrounding healthy cells [1]. Starting from specific histology dyes capable of staining certain cell types, the 20th century yielded achievements that led to sophisticated tools for chemotherapeutic treatment. Today’s strategies to achieve Ehrlich’s vision include the use of bioconjugates of drugs tethered to targeting agents. Therapeutic oligonucleotides (ONTs) are a class of drugs that are appealing for such applications as they allow alteration and control of abnormal gene expression patterns in cells affected by diseases [2]. Additionally, given that they successfully enter target cells, they are not as limited as proteins whose activities can be inhibited by antibodies or small molecules (e.g., hormone receptors, enzymes, or ion channels). However, systemic administration of ONT therapeutics such as siRNAs, microRNAs (miRNAs), and antisense ONTs bears several biological hurdles that need to be overcome. One of these obstacles is that due to their polyanionic characteristics, nucleic acids cannot penetrate cells because of poor transit across lipid bilayers. Various adjuvants such as lipids or liposomes [3], peptides [4], cationic polymers, or nanoparticle assemblies [5] have been tested that shield the anionic charges to improve uptake into target cells.

Alternatively, cell-specificity can be enhanced by conjugation of a ligand that enables active binding to a cell-surface protein on the target cell. Naturally occurring or artificial ligands have been explored, as such active carrier molecules that direct a siRNA to the active site within the patient body. Examples of natural targeting moieties include hydrocarbon structures being recognized by lectins on target cells (e.g., GalNac binding to asialoglycoprotein receptors (ASGPR) present on the surface of hepatocytes) [6]. Bioconjugates with transferrin (Tf), for transcytosis through the blood-brain barrier (BBB), have been used to deliver siRNAs to the brain [7,8]. Triblock polymer nano-assemblies carrying siRNAs exhibit increased targeting properties to various cancer cells when decorated with folate units (or other vitamins actively endocytosed by target cells), thereby facilitating binding to folate receptor overexpressed on cancer cells [9]. The advantages and disadvantages of the different delivery strategies are debated in the field. Major concerns are (1) extra- and intracellular hurdles for accessibility of the target cell’s cytosolic compartment (e.g., degradation, elimination from vasculature or renal excretion) and (2) side effects due to molecular interactions with other cells or serum biomolecules (e.g., immune response, platelet aggregation upon charge interaction, accumulation in the liver). Two recent review papers are recommended to the reader, summarizing the pitfalls in active drug targeting and to which extent the exploited carrier materials can overcome them [10,11].

**Figure 1:**
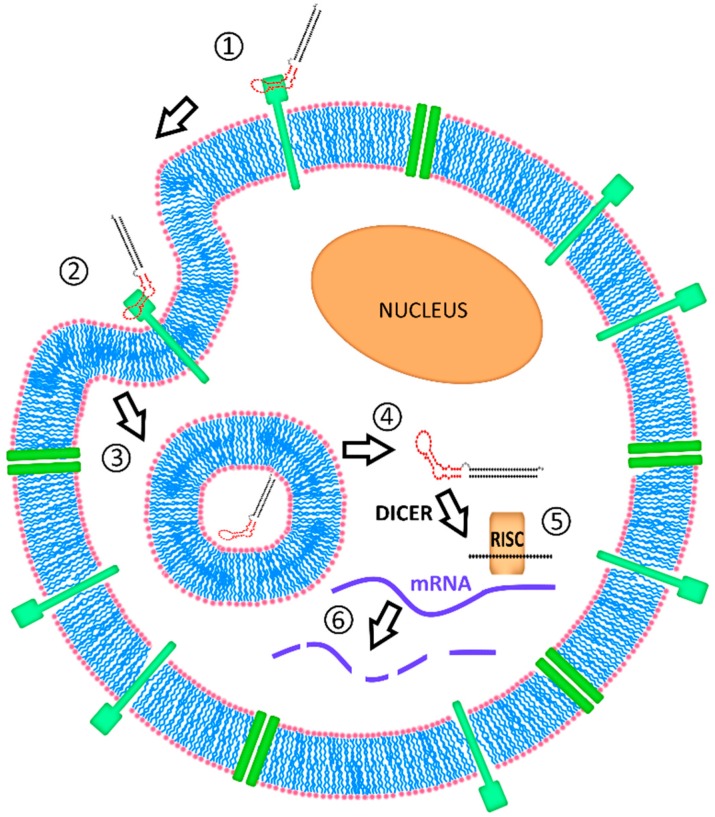
Concept of aptamer siRNA chimera (AsiC) delivery into a target cell. The AsiC molecule binds through the aptamer part (red) to the target molecule (in green, e.g., a cell-specific receptor) on the cell-surface (1); Upon endocytosis, AsiC molecules are internalized into the cell (2); where they, at least temporarily, end up in vesicles of the endosome or lysosome (3); Molecules escaping the endosomal compartment (4) will be recognized by RNA interference (RNAi) machinery. The ribonuclease Dicer will bind to the siRNA part of the AsiC (black double strand), cut it off the aptamer and load it into the RNA-induced silencing complex (RISC), an assembly of proteins further mediating the specific degradation of messenger RNAs (mRNAs) (5); The Argonaute protein AGO2, one major component of the RISC, unwinds the siRNA double strand and retains only one of the two strands, the guide strand. As a result, mRNAs matching this RISC-loaded guide strand can be cleaved, and the expression of the gene corresponding to this mRNA will be decreased (6).

Aptamers are artificially selected cell-specific ONT ligands that allow targeting to a broad range of cells. Aptamers are short single-stranded ONTs with recognition properties for certain target molecules due to their unique three-dimensional structure. High affinity and specificity for target molecules make them similar to monoclonal antibodies. However, aptamers possess some advantages over antibodies, including little-to-no immunogenicity and toxicity [12], longer shelf-life, lower production costs, and low batch-to-batch variation. They have been exploited for targeted delivery into a variety of cells and in combination with various drugs, including siRNAs [13]. Figure 1 outlines the delivery of an siRNA carried by an aptamer into a target cell.

The following sections will provide an overview of the current knowledge on the general application of aptamer-siRNA chimeras (AsiCs) and conjugates. We will review the different conjugation strategies described in the field, the advantages and limitations for therapeutic approaches, and the most recent attempts using AsiCs and aptamer-siRNA conjugates for the treatment of viral infections and cancer.

## 2. Aptamers as Ligands for the Targeted Delivery of Therapeutic Oligonucleotides

### 2.1. Aptamer Development and Identification

**Figure 2:**
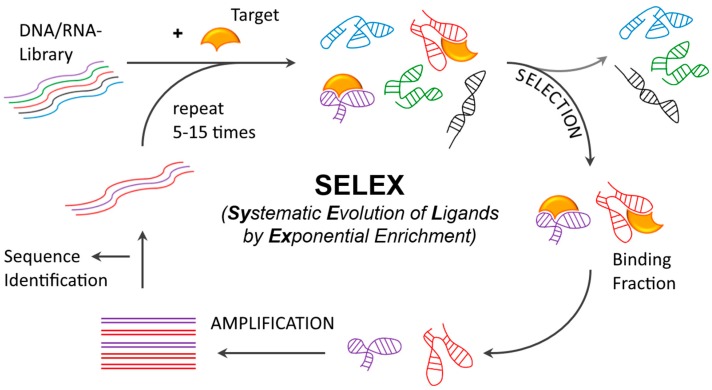
Systematic Evolution of Ligands by Exponential Enrichment (SELEX). Generation of aptamers is performed by the iterative selection of binders from a starting pool. The starting pool contains RNA or DNA oligonucleotides comprising constant terminal regions for primer annealing and a randomized central region (typically 20–50 nt). Upon exposure to the target of interest binders are separated from the nonbinding fraction (Selection). A counter-selection step using matrix only or a negative cell line in the case of Cell-SELEX is normally applied in addition, either before or after the selection step using the target, to eliminate non-specific binders. After elution of the bound fraction from the target molecule, real time-PCR (RT-PCR) (RNA) or PCR (DNA) is applied (Amplification). Single-strand synthesis leads to an enriched oligonucleotide pool, which is repeatedly subjected the selection procedure. Potential aptamer sequences are picked by sequence analysis of the final pool or even after every round of selection, mostly by means of next generation sequencing (NGS). Predominant candidates will be examined for their target affinity.

Systematic Evolution of Ligands by Exponential Enrichment (SELEX) [14,15,16], the original in vitro selection method used for selecting aptamer ligands to small molecules and recombinant proteins, can be modified to yield aptamers capable of binding to a variety of different targets including malignant or infected cells. This modified SELEX methodology, coined “cell-SELEX”, includes iterative steps designed to capture aptamer candidates from the complex ONT library against a surface marker protein which, is selectively expressed on the target cells. Separation of the unbound library fraction is usually achieved by immobilization of the target to a solid phase (e.g., ferromagnetic beads or cells on plates) or by capturing it after exposure to the library (e.g., filtration through a nitrocellulose membrane binding the target protein, or lysis of cells to capture cell-internalized aptamers). The bound library fraction is then amplified by polymerase chain reaction (PCR) after each selection step to ensure a sufficient amount of material for the subsequent selection step. Eventually, sequence and functional (measure of binding to target) analysis with individual sequences from the selected pool (after ~5–15 selection rounds) will yield aptamers with the desired characteristics. During the selection process, stringency (or selective pressure) is typically increased to favor the identification of sequences with the desired characteristics (ex. high affinity binders with high on-rates and slow off-rates). Here, it is worth noting that in early selection rounds, an excess of target molecules sufficient to provide the theoretical chance of binding to every library molecule should be used. The ratio of target molecules to ONT library molecules should be decreased gradually in subsequent selection rounds to favor the isolation of highly competitive sequences. Also, increasing the number and length of the wash steps will ensure that high affinity binders with slow off-rates can be selected. (Figure 2).

If the identity of the cell-surface protein is not known, SELEX can also be performed by using whole cells as targets (Cell-SELEX) [17,18], also ensuring that aptamers capable of binding to the native conformation of the target protein accessible on the cell-surface are obtained. Thus, whole cell selection also offers preferable advantages for selection against a known target protein. However, using whole cells as the carriers of the target protein generally leads to increased background binding. Moreover, cell viability should be monitored, as dead cells tend to bind nucleic acids non-specifically. To overcome this problem, divalent metal ion chelators such as ethylenediaminetetraacetic acid (EDTA) have been used during the recovery step [19]. The rationale behind this is that specific binders being stabilized in their secondary and tertiary structure will be removed from the target, while non-specific binding tends to occur by charge interactions of unstructured ONTs. To ensure aptamer binding specificity, an additional counter-selection step using a non-target cell line (ex. cells which do not express the cell-surface receptor or non-malignant cells in the case of cancer cell selection) can be performed at every cycle, either before the target enrichment step or after [18,20,21]. For a known target protein, negative cells can be produced using different methods. Typically, cells exogenously overexpressing the target gene are used as the target cells, while the same wild type cells not expressing the target gene serve as negative cells [22]. Alternatively, negative cells can be engineered by silencing the expression of the target gene using RNA interference (RNAi) gene knockdown [23] or clustered regularly interspaced short palindromic repeats (CRISPR)-Cas9 gene editing [24]. Also, completely unrelated cells or non-malignant cells of the same tissue are commonly used for the counter-selection step [17]. While in early applications the final step of sequence identification was carried out using molecular cloning, the development of next generation sequencing (NGS) methods has enabled fast and comprehensive analysis of all rounds, thus enabling investigators to closely monitor the progression of the selection from Round 0 to the final round, for each individual aptamer sequence [25,26]. Reviews on the implication of this method and further advances made in the aptamer selection process are recommended to the reader [17,27,28].

Variations on the selection process, (1) in vitro-SELEX (recombinant proteins); (2) whole cell-SELEX (cells); (3) ex vivo-SELEX (tissues) or (4) in vivo-SELEX (live animals), can be combined, taking advantage of the benefits of each method. Depending on the target, it can be beneficial to use a sequence of recombinant protein selection followed by cell selection [29] or vice versa [30]. Cell-internalization SELEX has been reported by Thiel et al. to generate aptamers suitable as drug delivery tools [31]. In this protocol, only endocytosed nucleic acids from the library are carried over to the subsequent selection step. Another method for selecting only internalized aptamers was reported in 2016 by Mu et al., and was termed “Conjugate-SELEX” [32]. Here, the authors conjugated the aptamer library to liposomal nanoparticles (LNP) and selected and applied the LNP-conjugated aptamer library to human head and neck squamous cell carcinoma cells. Those cells that had taken up the LNPs were separated from the LNP negative cells and processed to enable the identification of the cell-internalized aptamers.

Ex-vivo-SELEX was performed to favor the isolation of aptamers for targets in the context of whole tissues/organs or fixed tissues. Li et al. reported on an aptamer binding to heterogeneous nuclear ribonucleoprotein (hnRNP) A1 [33]. In this study, the DNA-aptamer BC15 was obtained from a selection set-up using paraffin tissue sections from infiltrating breast cancer carcinoma. Its target, hnRNP A1, was identified after the selection, using magnetic beads enrichment and mass spectrometry for identification. In a more recent paper, Wang et al. presented the concept of Morph-X-Select, a morphology-based tissue aptamer selection [34]. In this variation, instead of whole tissue sections, only regions of interest identified by morphological assessment, dissected by means of image directed laser microdissections (LMDs), were used. The authors applied this method to select a thio-modified DNA-aptamer using ovarian tumor vasculature. The target of this aptamer (V5) was identified as tumor marker vimentin.

The first attempt to carry out in vivo aptamer selection was published in 2010 by Mi et al., selecting RNA aptamers for p68, an RNA helicase and a member of the DEAD-box family involved in RNA processing [35]. p68 is aberrantly expressed in tumor tissues, making it an ideal target for cancer therapy. RNA aptamers binding to p68 were obtained by using an animal model of intrahepatic colorectal cancer metastases. The 2′-F-pyrimidine (2′-F-Py) modified RNA-library was injected intravenously and the bound fraction was retrieved from harvested liver tumors. In a similar approach of in vivo-SELEX published in 2013, Cheng et al. selected RNA-aptamers with the ability to surpass the BBB [36]. While, in this case, the actual target was not determined, the authors hypothesized that the aptamer utilized a transcytosis pathway to bypass the BBB.

### 2.2. Early Aptamer-siRNA Conjugates

Aptamers and siRNAs are both nucleic acids. Thus, the combination of aptamers with siRNAs, for cell-specific drug delivery, seems to be reasonable, since conjugation can be achieved in a straightforward manner by either covalent linkage or complementation (annealing). Combinations of aptamers and siRNAs have been referred to as AsiCs or aptamer–siRNA conjugates. Aptamer-mediated delivery of siRNAs was first described in 2006, by two independent research groups. In both studies prostate-specific membrane antigen (PSMA) targeting RNA aptamers were used. The biotin-streptavidin construct designed by Ellington and co-workers [37] comprised two biotinylated A9 anti-PSMA aptamers and two biotinylated dicer substrate siRNAs (DsiRNAs) against lamin A/C or glyceraldehyde 3-phosphate dehydrogenase (GAPDH). RNAi activity was observed in PSMA-positive LNCaP cells (with PSMA-negative PC-3 cells as a control) upon treatment with this conjugate. Sullenger’s group [38] harnessed a different PSMA-aptamer, A10. The PSMA A10 aptamer was used to deliver siRNAs targeting polo-like kinase 1 (*plk1*) and *bcl-2*, two genes generally overexpressed in human tumors. In this study, the authors covalently linked the passenger strand of these siRNAs to A10’s 3’-terminus. The respective guide strand was subsequently annealed to the aptamer-siRNA oligo. The authors confirmed internalization, as well as RNAi-mediated silencing of the siRNA target genes resulting in cancer cell death. Furthermore, in a human xenograft mouse model of prostate cancer, decreased tumor growth and tumor regression was observed. This study was the first to describe in vivo efficacy of aptamer-siRNA oligonucleotides.

### 2.3. Conjugation Strategies

Compromised siRNA activity and lack of economic feasibility in the production of longer RNAs (>100 nt) led to of the design of various aptamer conjugates. In 2009, the Giangrande group described optimizations of their original PSMA AsiC (A10-Plk1) to increase targeting specificity and silencing potency of the RNA drug. This was the first demonstration of efficacy upon systemic administration of an AsiC [39]. After successful aptamer truncation 3′-overhangs (UU) were applied to increase dicer enzyme recognition. Next, a wobble base pair at the 3′-terminus of the passenger strand was tested, to favor loading of the correct strand (guide) into the RNA-induced silencing complex (RISC). In further constructs, the authors swapped the siRNA strands within the AsiC, or designed the AsiC as one stem-loop-RNA. These two versions yielded the most pronounced RNAi effect. The in vivo circulating half-life was increased from <35 min to >30 h by addition of polyethylene glycol at the 5′-end (PEGylation). The PSMA aptamer A10 and its truncations have been further combined with several other siRNAs by various research groups [40]. One design was a bivalent PSMA aptamer AsiC with a small hairpin RNA (shRNA) against eukaryotic elongation factor 2 (EEF2) that exhibited up to four-fold greater cellular uptake than an analogous monovalent construct [41].

Berezhnoy et al. analyzed how siRNA silencing ability is affected by the conjugation to an aptamer. The authors used AsiCs published by other groups as examples for their study [42]. This study revealed that the degree of impairment is dependent on the thermal stability of the siRNA, with siRNAs having lower melting temperatures being less affected by conjugation to an aptamer. Furthermore, which end of the aptamer the siRNA was appended to also seemed to affect silencing. Conjugation of the siRNA to the 3′ end of 2′-F-Py RNA-aptamers, showed the lowest reduction of the RNAi effect. Finally, it was found that if an aptamer was co-transcribed with the passenger strand on the 3′-terminus and 3′-overhangs were removed on the passenger strand, the AsiC retained full activity (comparable to unconjugated siRNA duplex).

Chemical conjugation has also been described in the context of an aptamer-siRNA nano-assembly complexes [43,44]. Conjugation strategies have included the use of acid labile or redox sensitive linkers [37,45]. These linkers can be cleaved to release the siRNA from the conjugate in the acidic environment of the endosome (pH 4.5–6.8) [46] or upon the presence of oxidoreductases such as protein disulfide isomerases (PDI).

Another method for conjugating aptamers to siRNAs involves the use of a “universal” linker. For this approach, the aptamer and siRNA sequences are extended to contain complementary sequences that can be annealed to form a “sticky-bridge”. This conjugation method was first described by Rossi and co-workers and is discussed in more detail below, in the chapter on anti-viral therapy [47]. Kissing loop interactions, as found naturally in the packaging RNA of bacteriophage phi29 were also used [48,49], as well as complex nucleic acid origami assemblies [50]. Further interactions exploited for conjugation are electrostatic charges [44], such as in nano-assemblies [43], and pairs of non-covalently binding molecules, such as the biotin-(strept-)avidin interaction [37].

## 3. Recent Advances in Aptamer-siRNA Applications

### 3.1. Cytotoxic Cancer Therapy

As mentioned above, PSMA is one of the first cancer cell-specific marker proteins used for siRNA delivery through aptamers. Other cancer cell-specific aptamer targets exploited for AsiC experiments or in combination with other drugs include receptor tyrosine kinases (RTKs) such as epidermal growth factor receptor (EGFR; detected on glioma, lung, and breast cancer) [51,52], human epidermal growth factor receptor 2 (Erbb2 or HER2; particularly used for targeting breast cancer cells) [20], and tyrosine-protein kinase-like 7 (PTK7; overexpressed in many different cancers) [53]. For highly metastatic cancers, an aptamer recognizing alpha V and beta 3 (αVβ3) integrin was selected and joined to a siRNA against eukaryotic elongation factor 2, inhibiting proliferation and inducing apoptosis in target cells [54]. Furthermore, also used as targets were an atypically glycosylated form of mucin (MUC-1) overexpressed on various human adenocarcinomas [55], extracellular matrix protein Tenascin-C [56], and nucleolin [57], a protein almost universally present on cancer cell-surfaces and shuttling into the nucleus where it is found solely in benign cells. However, since the present paper focuses on more recent achievements and trends, we would like to point out other reviews covering earlier approaches for these aptamers [13,45,58,59].

**Figure 3:**
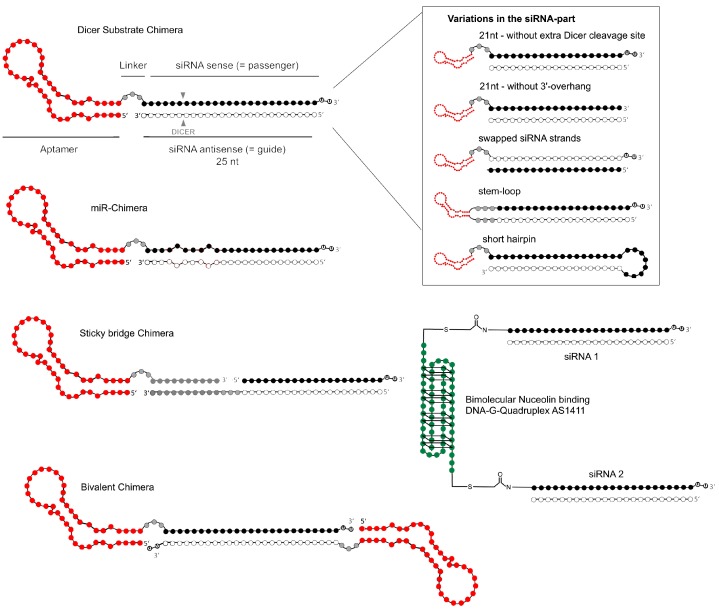
Aptamer-siRNA AsiC designs. Shown are different design strategies pursued. The Dicer-Substrate design as shown at the top, represents the most promising design for a universally functional combination of aptamer and siRNAs. The siRNA portion (21 nt) is appended through an extension of a 6 nt double strand, ensuring correct Dicer cleavage [47,62]. Adding a 2 nt 3′-overhang to the sense strand and choosing this strand as the one connected to the aptamers 3′-terminus is thought to lead to superior siRNA target knockdown. Other variations published (box on the right) comprise: A greater number of reported AsiCs, particularly in earlier studies, used 21 nt siRNAs lacking a Dicer substrate cleavage site [38]. Also tested were constructs lacking the 3′-overhang, swapping the strands in the siRNA, or appending both siRNA strands to the aptamer-RNA, creating a stem-loop construct [39]. Wullner et al. reported a AsiC using a short hair-pin RNA (shRNA) design [41]. AsiCs with miRNAs implemented instead of an siRNA have also been reported [63,64,65]. A convenient way of testing multiple siRNA adapters on an aptamer and preserving the RNAi potential was presented by the sticky bridge design, in which aptamer and siRNA part are annealed through a reverse complementary pair of strands [66]. Constructs consisting of two aptamer units (bivalent AsiC) [41] or two siRNAs on the bimolecular nucleolin DNA-aptamer [60] have been shown to benefit from increased affinity or synergistic knockdown effects, respectively.

The nucleolin aptamer AS1411 has been used as a AsiC with an siRNA that exhibited two unprecedented features [60]. First, this AsiC displayed an example of a DNA-aptamer conjugated to an siRNA, linked by non-cleavable maleimide chemistry. Secondly, since AS1411 is composed of two separate DNA-strands of the same G-rich sequence (each 25 nt) that together fold into a G-quadruplex structure [61], the AsiC was able to carry two siRNA units, one on each of the two DNA strands (Figure 3). This AsiC was specifically designed to suppress tumor invasion and angiogenesis by choosing siRNAs targeting the mRNAs of two genes involved in metastasis-associated epithelial-mesenchymal transition (EMT), snail family zinc finger 2 (SLUG) and neuropilin 1 (NRP1), which promote malignant transformation and activate key signaling pathways during different stages of metastasis.

Nucleolin is a multifunctional hnRNP present in the nucleus, but also in the cytoplasm and on the cell-surface, shuttling between these compartments. Additionally, nucleolin is upregulated in highly proliferating cells, including breast cancer, lymphocytic leukemia, and prostate carcinoma [67,68]. To evaluate the efficiency of the AsiC, an in vivo lung cancer model was established by inoculation of non-obese-diabetic severe-combined-immunodeficiency (NOD SCID) mice with CL1-5 cells. These animals were then treated with each of the two AsiCs alone, or a combination of the two (a combination of 0.5 dose equivalents of the single AsiC treatment) intratumorally three times per week for 42 days. The tumor growth rate decreased by three-fold when single AsiCs or a combination of both AsiCs were administered. Additionally, the combination of the two AsiCs exhibited a synergistic effect, suppressing tumor invasion.

Recently, Wang et al. used AS1411 as a targeting decoration on cell-derived micelle-like vesicles (termed as extracellular vesicles) to deliver siRNAs and microRNAs (mRNAs) into MDA-MB-231 breast cancer cells [69]. AS1411 potentially inhibits tumor growth [57,70]. Thus, this aptamer has been used in three Phase II clinical trials and is one of the most promising candidates for approval by the U.S. Food and Drug Administration (FDA).

In another study, a co-delivery of two siRNAs in a bivalent PSMA aptamer (A10-3.2) AsiC was reported (see Figure 3) [71]. To tackle metastatic castration resistant prostate cancer (mCRPC), the authors chose EGFR and survivin as the targets for siRNA silencing. EGFR overexpression is associated with mCRPC and bone metastasis frequently occurring in advanced stages [72,73]. Survivin is known as a member of the inhibitor of apoptosis protein (IAP) family and, thus, plays a pivotal role in the progression of PCa and other solid tumors [74]. The AsiC was tested in a C4-2 PCa xenograft model, where it significantly suppressed tumor growth and angiogenesis. As confirmed by rapid amplification of cDNA ends (5′RACE) PCR, the inhibition of angiogenesis was mediated by an EGFR-dependent mechanism.

A significant portion of cancer-related genes is regulated by miRNAs, and many reports demonstrated that miRNA expression is deregulated in human cancers [75,76]. Thus, miRNA delivery has garnered attention within the past years, and the restoration of miRNA levels by specific delivery tools represents one strategy for cancer therapy. In 2014, Esposito et al. reported a multifunctional aptamer–miRNA construct for myeloid leukemia therapy [63]. The AsiC construct (GL21.T–let) was composed of the RNA-aptamer GL21.T and the tumor-suppressing miRNA let-7g. GL21.T binds to oncogenic RTK Axl (*K*_d_ = 12 nm) and led to abrogation of Axl-dependent signal transduction, such as extracellular-signal regulated kinase (ERK) and protein kinase B (PKB, Akt) phosphorylation [77]. For Axl-positive cells in cell culture treatment with GL21.T–let, cancer cell survival and migration was strongly reduced. Also, inhibition of tumor growth was observed in a xenograft mouse model of human lung cancer. Recently, the GL21.T aptamer was used to deliver miR-212 into human non-small cell lung cancer (NSCLC) cells [64]. This AsiC can inhibit the anti-apoptotic phosphoprotein enriched in diabetes/phosphoprotein enriched in astrocytes (PED/PEA-15) implicated in a common treatment resistance against TNF-related apoptosis-inducing ligand (TRAIL). Using this approach, NSCLC cells were sensitized to TRAIL therapy and exhibited increased caspase activation. Recently, RNA-aptamer GL21.T was converted into a more stable DNA-aptamer with 2′-F-Py modifications and 5′-phosphorothioates at certain positions [78]. This aptamer was examined for its ability to block Axl-phosphorylation in ovarian cancer using intraperitoneal animal models. Impairment of tumor growth and reduction of metastatic nodules was observed along with inhibition of migration and invasion of the cancer cells. Carla Esposito and Vittorio de Franciscis also identified an aptamer to the insulin receptor (IR), named GL56, using cell-internalization SELEX [79]. Based on its ability to undergo efficient and rapid cell-uptake, this aptamer is a promising tool for the delivery of small RNAs into IR-dependent cancer cells.

### 3.2. Cancer Stem Cell Therapy

Cancer cells can be subdivided into different types based on their occurrence, morphology, behavior, and potential to evade natural defense or differentiate into another cell type. Cancer cells which exhibit the two lattermost abilities, are referred to as cancer stem cells (CSC), as they share capabilities like the ones ascribed to stem cells. They reproduce themselves and sustain the tissue or tumor. Targeting CSCs has therefore become a central interest in the development of new therapies. Moreover, new drugs are urgently needed as these cells show intrinsic resistance to conventional treatments [80]. CSC specific biomarkers are required for targeted delivery using aptamers. Researchers in the field have been focusing on improving the cell-SELEX protocol to select aptamers predominantly for CSCs [81]. Cell-surface markers such as the epithelial cell adhesion molecule (EpCAM), CD44, and CD133 have been tested for aptamer-mediated cell therapy [82]. Other CSC-specific targets have been suggested, including CD34, a regulator of cell adhesion [83]. Furthermore, CD38 is normally expressed on hematopoietic cells. Greater expression levels are found in bone marrow precursor cells protecting germline cells against apoptosis. While it is lost in mature B-cells, it is found in cells of chronic lymphocytic leukemia (CLL), where its expression signifies poor prognosis [84]. Besides, CD38 was also shown to be expressed in skeletal and heart muscle, proximal convoluted tubules of kidney, and normal adult prostate [83,85]. Whether cells of these tissue types would also be influenced by utilization of an aptamer targeting CD38 remains unclear. However, recent findings have shown, that deficiency or inhibition of CD38, which can degrade different nicotinamide dinucleotides, led to positive prognostic effects in cardiac tissues, such as protection against ischemia and reperfusion injury [86] and endothelial dysfunction [87]. Additionally, CD44 and CD24, normally expressed on B-cells, have been discussed as CSC markers for many carcinomas, with an emphasis on breast cancer [88,89]. CD90 has been suggested as a marker of CSCs from the brain [90], liver [91], gastric [92], and lung tumors [93].

Shigdar et al. presented RNA-aptamers selected for CD133, out of which one (CD133-A15) was truncated to a 15mer [94]. Endocytosis of the CD133-aptamers by HT29 colon cancer cells was confirmed by confocal microscopy, while for five different negative cell lines, no binding or internalization was observed, confirming the specificity of CD133-A15. Shortly after, Jiang et al. used this aptamer to deliver salinomycin-loaded nanoparticles into CD133+ hepatocellular carcinoma (HCC) cells, inducing apoptosis [95]. Meanwhile, Chen et al. targeted dysfunctional epithelial progenitor cells (EPCs) instead of CSCs using CD133-A15 in a AsiC with a siRNA targeting adenosine kinase (ADK), showing the potential of this very small aptamer for AsiC delivery [96].

EpCAM is overexpressed on tumor initiating CSCs, and thus aptamers binding to this molecule have been exploited for the delivery of siRNA AsiCs. In 2015, three groups reported on different approaches using EpCAM aptamers for delivery of siRNAs. Under the leadership of Wei Dun and Sarah Shigdar, a chemo-sensitizing approach using a Dicer substrate siRNA against survivin appended to an 18mer anti-EpCAM RNA-aptamer [97] in a breast cancer xenograft mouse model was presented [98]. Doxorubicin resistant MCF-7/ADR cells, in which survivin expression is 21-fold that of progenitor cells, were generated and subsequently used in the xenograft model. Administration of the AsiC to mice reversed tumor cell chemo-resistance, and a low dose of doxorubicin inhibited cell stemness as documented by the expression of different markers, eliminated cancer stem cells via apoptosis, and suppressed tumor growth, leading to prolonged survival of mice bearing chemo-resistant tumors.

The second paper published in 2015 described the use of an EpCAM aptamer linked to a PlK-1 siRNA for the treatment of breast cancer [99]. In this study, tumor initiating triple-negative breast cancer (TNBC) cells (cells negative for estrogen receptors, progesterone receptors, and HER2) were targeted. The growth of Basal-A TNBCs (resembling basal-A TNBC primary tumors) was reduced upon subcutaneous treatment with the AsiC in a mouse model as well as in human breast cancer tissues in vitro. Growth of normal epithelial cells, basal-B TNBC cell lines (which resemble mesenchymal TNBC primary tumors), or normal human breast tissues was not inhibited by the AsiC. The knockdown was proportional to EpCAM expression. Moreover, the AsiC-induced knockdown of the mitosis regulator PlK1, suppressed tumor-initiating cells (TICs) of epithelial breast cancer cells as shown in in vitro functional assays (colony and mammosphere formation).

Finally, in 2015, Krishnakumar and colleagues described a two-pronged approached to EpCAM inhibition in cancer cells. This group constructed an AsiC composed of an EpCAM binding aptamer and a siRNA targeting EpCAM mRNA [100], thereby creating a feedback loop in which the inhibition in the cancer cells was directly proportional to EpCAM expression. The anti-tumor activity was tested by using MCF7 cells in a mouse xenograft model. The AsiC induced EpCAM downregulation and inhibited cell proliferation. Different markers of pluripotency were downregulated upon this treatment: The transcription factors sex determining region Y box 2 (SOX2), octamer-binding transcription factor 4 (OCT4), and NANOG (not being an abbreviation but named after an old Celtic myth), as well as CD133. The same laboratory constructed polyethyleneimine (PEI)-based nanocomplexes (198 nm in diameter) bearing the same siRNA and aptamer against EpCAM [101]. Specific binding and uptake were demonstrated in cultured MCF7 cells and a retinoblastoma cell line (WERI-Rb1), but the in vivo efficacy of this construct still needs to be examined.

Recently, de Franciscis and colleagues reported an aptamer-mediated combinatorial miRNA delivery approach, targeting glioblastoma stem-like cells (GSCs) [65]. The authors constructed two RNA-aptamers with affinity to RTKs, one binding to Axl (GL21.T), which the authors had previously used to delivery microRNAs to cells, and another aptamer (Gint4.T) binding to Platelet-derived growth factor receptor beta (PDGFRβ). Both aptamers inherently act as inhibitors of their target RTKs, therefore augmenting the anti-tumorigenic effect. The combination of the tumor-suppressor, miR-137, and the antagonist of oncomiR, miR-10b, appended to the aptamers was tested and showed enhanced impact on tumor cell viability and migration. Furthermore, a reasonable amount of the aptamers and their respective AsiCs could cross the BBB. The authors speculated that this was a consequence of the transcytosis of the target RTKs. Interestingly, the Gint4.T aptamer has recently been conjugated to a mimetic peptide for targeted delivery to cardiac cells demonstrating the broad applicability of these aptamer ligands (personal communication).

### 3.3. Cancer-Immunotherapy

Immunotherapy is a rapidly expanding field in targeted cancer treatment. Instead of targeting the cancer cell itself, the agents used target immune cells. Historically, the idea of boosting the immune system to fight tumor growth goes back more than hundred years ago when, in the late 19th century, a New York surgeon, William Coley, attempted to stimulate immune responses by injecting bacteria into tumor sites of his patients.

Checkpoint blockade is one strategy of cancer immunotherapy. In 2003, the Gilboa group published the first aptamer acting as a checkpoint blocker by binding to cytotoxic T-lymphocyte associated protein 4 (CTLA-4), also known as CD152 [102]. CTLA-4 expressed on the surface of T-cells downregulates responses to stimuli such as the ones mediated by CD28 activation to prevent immunological overreaction [103]. The antagonistic RNA aptamer to CTLA-4 was arranged as a tetrameric construct which, due to multivalence-related effects, had enhanced potency in inhibiting CTLA-4 in vitro and tumor immunity in mice. Recently, a siRNA-AsiC using a CTLA-4 aptamer has been reported for the delivery of anti-STAT3 siRNAs to malignant T lymphocytes. STAT3 supports tumor survival, proliferation, and invasion and can lead to immunosuppression. An earlier study by Kortylewski et al. using CpG ONTs bound to Toll-like receptor-9 (TLR9) to deliver siRNAs indicated that STAT3 inhibition leads to antitumor immune response [104]. In addition, Herrmann et al., using the CTLA-4 aptamer-STAT3-siRNA AsiC in CD4+ T regulatory cells, showed that knocking down STAT3 along with the blockade of CTLA-4 caused an increase of CD8+ T effector cell response (and therefore increasing the impact of T lymphocytes against tumor cells) in an in vivo model [105].

Most cancer immunotherapies work by modulation of lymphocyte co-receptors, which can be inhibitory or stimulatory. 4-1BB (CD137/TNFSF9) is a stimulatory receptor found on various types of immune cells [106]. One of 4-1BB’s functions is the activation of CD8+ T-cells. An aptamer binding to 4-1BB acts as an artificial ligand inducing oligomerization of the receptor and, hence, initiates stimulatory signals, leading to increased T-cell survival [107].

The same 4-1BB aptamer was used by Berezhnoy et al. to deliver a siRNA against mTOR complex 1 (mTORC1) into CD8+ T-cells [108]. The dual function of this AsiC ensured that activation of the T-cells and suppression of mTORC1 proceeded in parallel. Consequently, differentiation of the targeted T-cells into memory T-cells was more efficient and immunosuppressive side effects from mTORC1 inhibition in other cell types were avoided, as they occur by non-selective treatment with rapamycin. The enhanced antitumor immunity was shown in a mouse model, in which animals were immunized with irradiated B16 melanoma cells, treated with the AsiC (i.v.) or rapamycin (i.p.) the next day, and finally were subjected to an additional xenotransplant of the melanoma tumor cells 50 days later.

Recently, Rajagopalan et al. reported a monovalent 4-1BB aptamer, which is not activated by itself, for siRNA delivery into already activated CD8+ T-cells [109] to modulate the differentiation of these cells towards memory precursor effector cells (MPECs) rather than towards short-lived effector cells (SLECs), which would undergo apoptotic death after a short life span. This strengthening of the immunological memory was induced by using an anti-CD25 siRNA. Downregulation of CD25, which is expressed on the target cells, prevented interleukin-2 (IL-2) binding to CD25 and the subsequent differentiation into SLECs.

### 3.4. Anti-Viral Therapy

Besides cancer treatment, AsiCs have been designed and tested for the treatment of viral infections, mainly against infections by the human immunodeficiency virus (HIV). RNA-aptamers binding to the virion protein HIV-1 gp120 [47,110,111] as well as cluster of differentiation 4 (CD4) on the surface of HIV-infected T-cells [112,113] have been selected to hinder the interaction of these two proteins during the entry of HIV-1 into its target cells. Moreover, these aptamers have successfully been combined with siRNAs targeting viral genes. The region in the 9.2 kB RNA genome of HIV preferentially targeted with siRNAs is an exon that encodes two essential regulatory elements of HIV, the HIV trans-activator (tat) and the regulator of expression of virion proteins (rev).

A gp120 aptamer AsiC designed by Zhou et al. combined blocking of viral adhesion through gp120 to CD4, and knockdown of the viral regulatory proteins [47,110]. It specifically suppressed the replication of HIV-1 in a humanized mouse model. The AsiC construct used in this study can be described as the original 2′-F-RNA-aptamer 3′-terminally extended by the sense strand of the siRNA. A 2–4 nt linker between these two sequences was applied for flexibility and enhanced Dicer processing. The siRNAs’ antisense strand was simply annealed to this construct. Zhou et al. also investigated the efficiency of AsiCs with different length of the siRNA. When a 27 nt Dicer substrate siRNA was implemented instead of a classical 21 nt siRNA, the silencing effect was improved by 20%. The authors speculated that Dicer-generated 21–23 nt siRNAs might be incorporated more efficiently into RISC. In a following study, the efficacy of the chimer was further evaluated [111]. In HIV-infected humanized mice, the intravenously administered gp120-aptamer-siRNA AsiC led to a reduction in viral loads by several orders of magnitude. The aptamer itself was already capable of inhibiting HIV-1 infection, but, when used as an aptamer-siRNA AsiC, the duration of the inhibition was enhanced, due to target mRNA degradation that was validated by 5′RACE PCR. In these experiments, AsiCs of non-binding aptamer variants and siRNAs lacking the aptamer served as negative controls.

The other prominent target for aptamer-mediated HIV treatment is human CD4 on T-helper cells. In 2011, Wheeler et al. published an aptamer-siRNA AsiC with an aptamer selected earlier for staining CD4 positive cells [112,113]. In this AsiC study, a 21 nt siRNA that targeted the mRNA of C-C-motive Chemokine Receptor type 5 (CCR5), which is used by HIV-1 as a co-receptor for its entry into the host cell, was chosen. In addition, HIV-1 *gag* and *vif* genes were used as siRNA targets. Specific gene knockdown was not only observed in CD4+ T cells and macrophages, but the AsiCs were also tested on human cervico-vaginal tissue explants. The authors also administered gels containing the aptamer siRNA AsiC intravaginally to humanized mice. In this model of topical application, vaginal transmission of HIV to both the mice and the cervico-vaginal explants was significantly prevented. Moreover, this study showed that locally and topically applied AsiCs were less subjected to degradation than those systemically delivered as in the gp120-aptamer AsiCs, resulting in enhanced efficacy.

A year later, Kai and colleagues successfully converted this CD4 targeting 39mer RNA-aptamer into a DNA-aptamer and used it for a siRNA-AsiC to downregulate HIV-1 protease [114]. This is noteworthy because the secondary structure of RNA- and DNA-analogs are likely to differ significantly, due to their different ribose puckering. Furthermore, molecular interactions might further be impacted in the case of a DNA-analog because of missing hydroxyl- and fluoro-substituents (compared to 2′-F-Py modified RNA that is usually employed). In this study, binding and uptake of the DNA-aptamer-AsiC into CD4+ T cells was confirmed by microscopy using the fluorescently labeled AsiC. The inhibitory effect on the HIV-1 protease was examined by using quantitative RT-PCR (qRT-PCR) on T cells that had been transfected with the mammalian expression vector plasmid pcDNA-HIR-PR. Interestingly, the DNA-aptamer AsiC was even more potent than the RNA-aptamer counterpart. While the reason for this finding was not elucidated, investigations with other RNA-aptamers should be pursued, as it might lead to a better understanding of AsiC applications in general.

Rossi and colleagues, who are responsible for the biggest innovations in the field of anti-viral aptamer-siRNA-AsiCs, also added bioconjugation strategies to the AsiC field [13,47,66,110]. One objective when designing AsiCs is to simplify conjugation, while preserving siRNA efficacy [42]. The sticky bridge approach, mentioned above, is a convenient method of combining the two RNAs in a non-covalent manner, providing opportunities for testing various combinations of each RNA at a reasonable cost [47]. A general example of these sticky bridge AsiCs is given in Figure 3. The sticky bridge comprises a GC-rich complementary annealing sequence that is appended to both RNAs enabling the annealing of the strands to each other. Additionally, a three-carbon linker provides the flexibility to the bridged RNAs, ensuring Dicer processing of the siRNA.

## 4. Aptamer-siRNA Development: Major Considerations

### 4.1. Selection of siRNA

Several aspects should be considered for the successful delivery of siRNAs by means of cell targeting aptamers. First, choosing a suitable target gene to be knocked down and an appropriate target site on the mRNA for the siRNA are key requirements. Access to online tools and rich empirical data amount have simplified the identification of sequences capable of inducing sufficient mRNA degradation. One should take advantage of these sources for deciding on the optimal siRNA sequence (e.g., reviewing original publications, patents, and clinical trials; or make use of sophisticated algorithms that underlie siRNA finder on siRNA vendors’ webpages). Otherwise, knockdown of a target mRNA could be impaired because of poor accessibility due to secondary mRNA structure or RNA-binding proteins, poor Dicer processing, or inefficient loading of the guide strand into the RISC. For further advice on experimental design, we refer the reader to recommendations made in this field [42,62,115].

### 4.2. Aptamer Considerations

The same caution should be exercised when choosing the aptamer. The aptamer must fulfil the following criteria: (1) selectivity and specificity for the desired target cell type; (2) sufficient number of target sites on the target cells and rapid internalization into the target cells with a substantial fraction of the nucleic acid remaining intact inside of the cell; (3) applicability for the intended administration in terms of pharmacokinetics (PK; stability, and biodistribution) and pharmacodynamics (PD; e.g., toxicity and immunogenicity) [116,117,118]. PK and PD of AsiCs for systemic delivery will be different from those of AsiCs used for local or even topical application. While a greater number of published aptamers lack detailed information on PK and PD as more comprehensive in vivo studies are required for these assessments, criteria (1) and (2) have already been evaluated and/or meet the requirements in a lot of original publications on aptamers. Furthermore, these aspects as well as aptamer stability in the presence of nucleases from mammalian sera, can feasibly be examined in ex vivo experiments. These mammalian nucleases are the main reason why most RNA-aptamers bear the 2′-F-Py modification on the pyrimidine nucleosides. This provides protection against RNAse A superfamily, whose members cleave RNA 3′-terminal of pyrimidine nucleosides (uridine and cytosine) [119]. Within the SELEX process, 2′-F-modified nucleosides can be implemented using a mutant of the T7-RNA polymerase (T7-RNAP), namely T7-RNAP-Y639F [120]. Other than the fluoro-substituent, amino- and methoxy-groups have been tested at the 2′-position for protection against these ubiquitous RNAses [121]. However, as no enzyme capable of efficiently incorporating these modified nucleosides within the SELEX process is available, they have not been used as extensively as the 2′-F-Py for aptamers.

### 4.3. Intracellular Fate of Aptamer-siRNA Conjugates

When an appropriate aptamer is lacking, a selection can be carried out by using the protocol established in our lab, termed Internalization SELEX (please refer to the earlier sub-chapter “aptamer selection”) [31]. Hence, aptamer candidates that not only rapidly bind and internalize into the target cells, but also assure that a substantial fraction of the internalized aptamer is internalized in the cell without being degraded, a circumstance pivotal to the actual potential of an endocytosed siRNA, can be selected.

However, for almost all aptamers successfully applied in siRNA deliveries, the intracellular fate, particularly the required transition across lipid biolayers such as the escape from endosomal vesicles, remains elusive. Insights into the kinetics of intracellular trafficking and spatially and high-resolution real time monitoring as well as methods such as STimulated Emission Depletion (STED) microscopy [122], Structured Illumination Microscopy (SIM), and Single Molecule Localization Microscopy (SMLM) [123] have recently garnered attention. Wittrup et al. presented an approach for the visualization of endosomal release of lipid-formulated siRNAs in HeLa cells [124]. Their findings suggest that siRNA release occurs invariably from maturing endosomes within 5–15 min of endocytosis. This agrees with the rationale that the addition of a release agent such as membrane penetration peptides or lipids that will become active at a certain pH during the endosome maturation [125] is necessary to facilitate the transition of a bigger part of RNA-molecules from the endosome into the cytosol. However, whether sufficient endosomal escape is ensured in AsiC applications devoid of such agents remains unclear.

## 5. Conclusions and Future Perspective

Nucleic acid aptamers have emerged as promising reagents for enabling the cell-targeted delivery of RNAi-based bio-drugs. In this review, we have summarized advances in the aptamer selection technology that favor the identification of aptamers with the ability to efficiently bind to cell-surface receptors in the context of the cell membrane while simultaneously undergoing receptor-mediated internalization. These technical advances have enable researchers to explore the use of aptamers for delivering therapeutic oligonucleotides, such as siRNAs, to target cells in vitro and in vivo. The initial proof-of-concept studies demonstrating efficacy of aptamer-siRNA AsiCs (AsiCs)/conjugates in cancer cells in culture and in mouse models of cancer were reviewed. In addition, recent advances in aptamer-siRNA drug designs and extent of therapeutic applications were presented. Important considerations for optimal aptamer-siRNA drug design and potential challenges regarding the subcellular fate of these RNAs are also discussed.

The past two years have witnessed the approval of several oligonucleotide-based drugs for treating neurodegenerative diseases such as spinal muscular atrophy (SMA) [126] or muscular diseases such as Duchenne muscular dystrophy (DMD) [127]. These successes are likely to pave the road for future RNA or DNA bio-drugs for other disease conditions including cancer, cardiovascular diseases, viral infections and rare diseases. While the future of oligonucleotide-based drugs is bright, continued efforts in several key areas are needed before aptamer-siRNA drugs are approved for use in humans. One limiting factor for their translation is cellular uptake efficiency and specificity. While traditional aptamers were identified using recombinant purified protein targets, recent advances in the aptamer selection methods technology, which include stringent negative selection steps and performing selections on live cells in culture, have made it possible to develop better aptamers for cell-targeted delivery applications. These new aptamers are better equipped to recognize cell-surface receptors in their native milieu (cell membrane) and undergo cellular uptake. In addition, stringent negative selection steps have made it possible to develop aptamers with exquisite binding specificity, low off-target interactions and overall better safety profiles. For more information regarding the advances in cell-SELEX technology please refer to the following reviews [17,31].

An additional hurdle to the translation of aptamer-siRNA drugs is efficient delivery of the therapeutic siRNA cargo to the appropriate subcellular compartment, in this case, the cytoplasm. Cytoplasmic delivery of aptamer-siRNA AsiCs/conjugates is currently an inefficient process, where less than 1–5% of aptamer-siRNA molecules that are taken up by the target cells are thought to escape the endosomal compartment and engage into RISC. Modifications to the cell-internalization SELEX protocol such as the inclusion of a cell-fractionation step to enrich for cytoplasmic targeted aptamers, would enrich for aptamer sequences capable of undergoing endosomal escape and delivering siRNAs to the cytoplasm. Conjugation of existing cell-surface receptor targeting aptamers to cationic amphipathic peptides or functionalized nanomaterials could serve as an alternative approach to enhance cytoplasmic delivery [128]. However, a better understanding of the endocytic release mechanism for aptamer-targeted drugs is warranted before one can afford solutions to this problem.

Finally, a better understanding of “drug” formulation, PK/PD properties, and potential toxicities of aptamer-siRNA drugs is needed before these novel classes of bio-drugs can be translated into humans. Recent advancements in aptamer technology and lessons learned from failed attempts to translate aptamer drugs into humans promise to facilitate the development of aptamer-based drugs with superior safety and efficacy profiles. Future efforts in rational aptamer drug development and implementation promise to pave the way for the next-generation of targeted aptamer-based drugs for the treatment of inherently challenging diseases such as cancer, cardiovascular disease, and viral infections.
